# Supplementary figures and images for: A Markov chain approach for ranking treatments in network meta‐analysis
Source: Stat Med. 2020 Oct 26;40(2):451–64. doi: 10.1002/sim.8784 (PMC7821202; doi:10.1002/sim.8784)

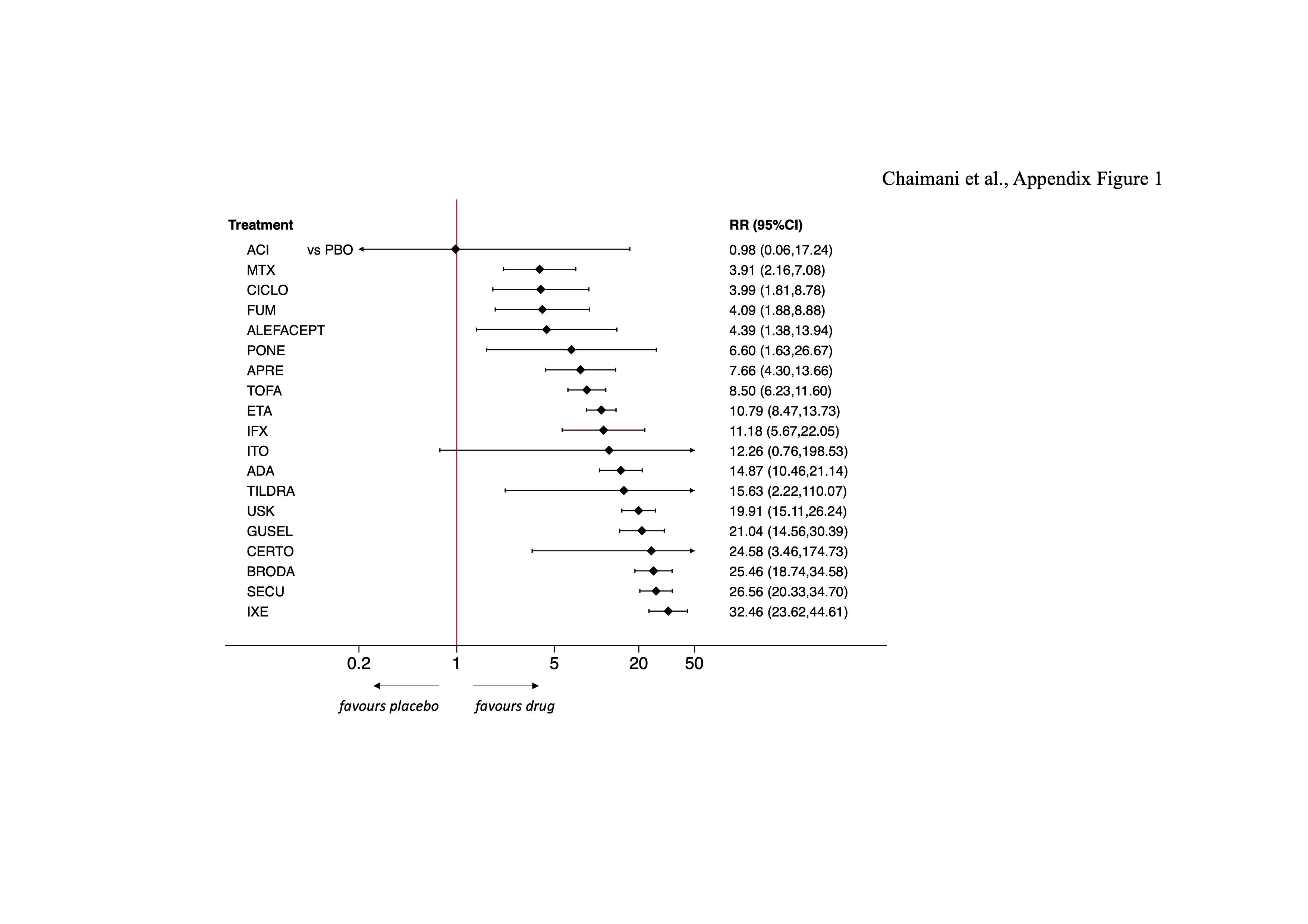

Supplement: Supplementary file 1 — Appendix Figure 1 Relative effects of all drugs vs placebo for efficacy. [file SIM-40-451-s001.tiff]

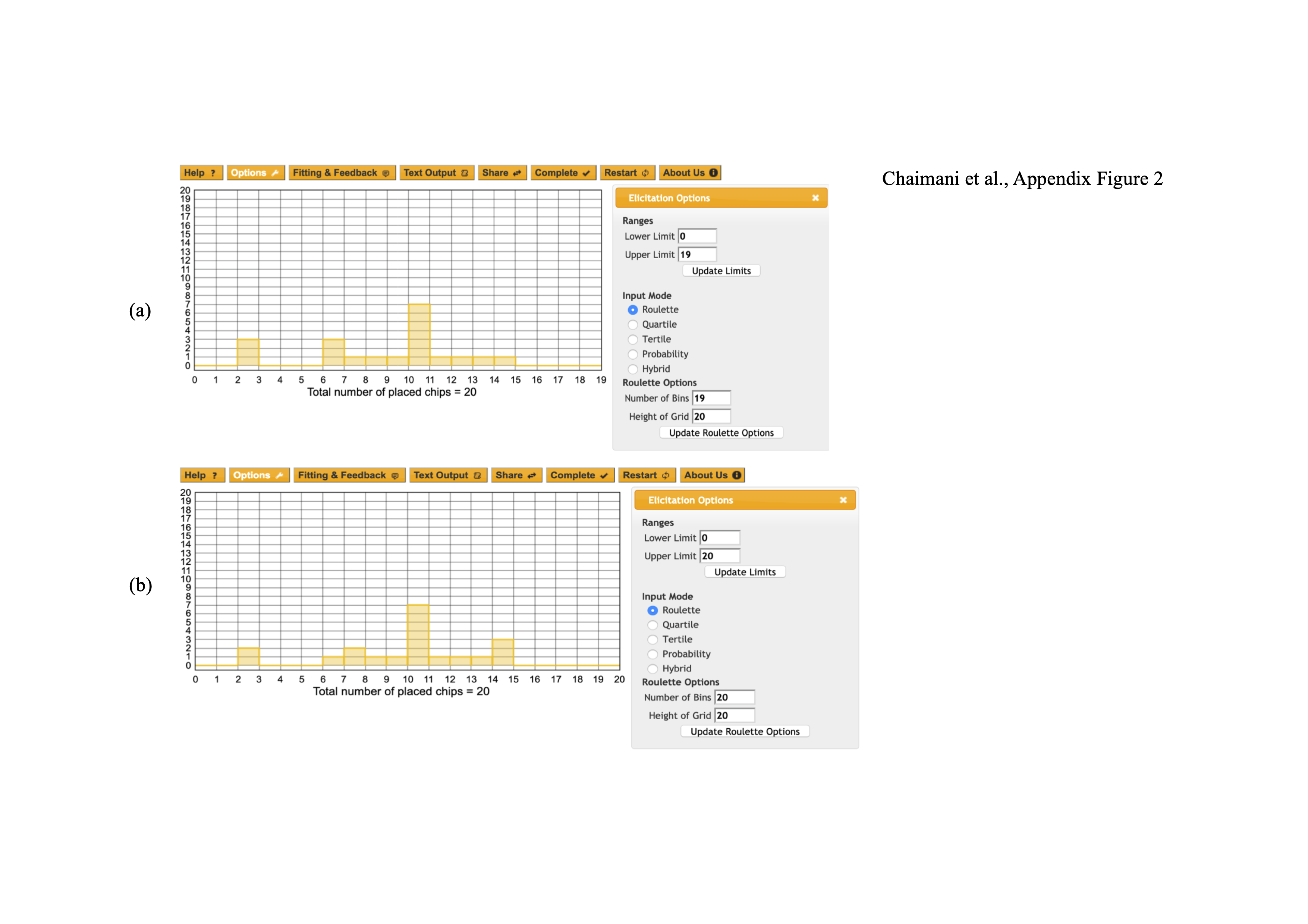

Supplement: Supplementary file 2 — Appendix Figure 2 Elicitation of the probabilities of selecting each treatment in the psoriasis network based on clinical experience for (a) efficacy and (b) safety. [file SIM-40-451-s002.tiff]

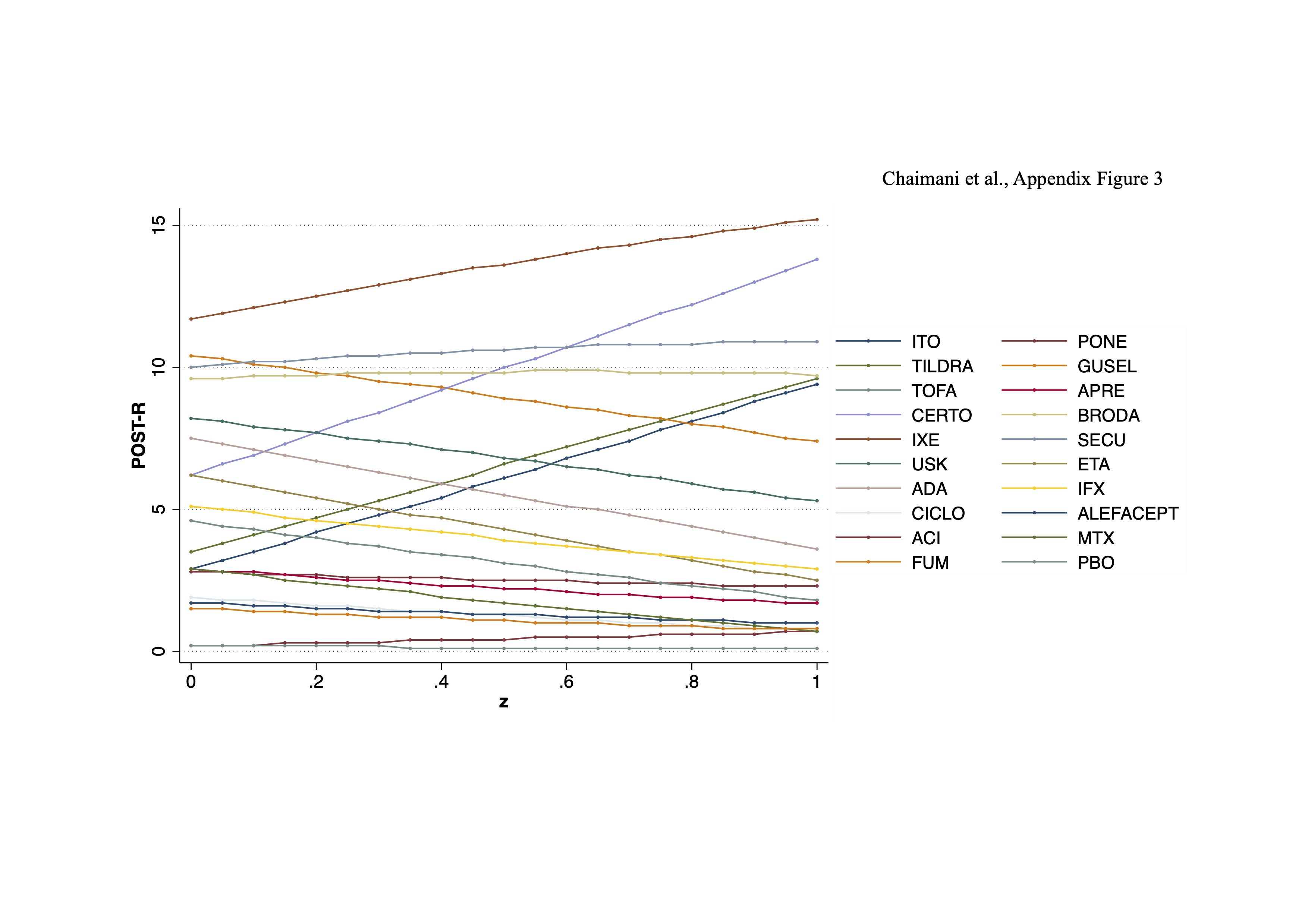

Supplement: Supplementary file 3 — Appendix Figure 3 Sensitivity analysis on a range of values between 0 and 1 for the probability z considering the confidence in the evidence along with the relative effects. Each dot corresponds to the POST‐R score of the respective drug for each z. [file SIM-40-451-s003.tiff]
