# Supplementary material for: A Markov chain approach for ranking treatments in network meta‐analysis
Source: Stat Med. 2020 Oct 26;40(2):451–64. doi: 10.1002/sim.8784 (PMC7821202; doi:10.1002/sim.8784)
Supplement: Supplementary file 4 — Appendix Table 1 League table for efficacy (PASI 90) in lower triangle and safety (serious adverse events [SAE]) in upper triangle. Each cell contains the risk ratio and the respective 95% confidence interval between the treatment in the column and the treatment in the row. Values larger than 1 favour the treatment in the column for both outcomes. Appendix Table 2. League table of probabilities pi > j for efficacy (PASI 90) in lower triangle and safety (serious adverse events [SAE]) in upper triangle. For both outcomes each cell contains the percentage probability that the treatment on the left produces a better outcome than the treatment on the right. Appendix Table 3. Confidence in the evidence for efficacy (PASI 90) as obtained by CINeMA. Appendix Table 4. Probabilities of trusting to use each treatment based on the confidence of the evidence in the respective pairwise comparison. Appendix Table 5. Information on treatment ranking for the psoriasis network in terms of treatment cost. Grey cells correspond to the five top positions. [file SIM-40-451-s004.docx]

**Appendix Table 1**. League table of relative effects for efficacy (PASI 90) in lower triangle and safety (serious adverse events (SAE)) in upper triangle. Each cell contains the risk ratio and the respective 95% confidence interval between the treatment in the column and the treatment in the row. Values larger than 1 favour the treatment in the column for both outcomes.

| **SAE** | | | | | | | | | | | | | | | | | | | | |
| --- | --- | --- | --- | --- | --- | --- | --- | --- | --- | --- | --- | --- | --- | --- | --- | --- | --- | --- | --- | --- |
| **ACI** | 0.97 (0.02,49.98) | 1.37  (0.03,73.58) | 1.18 (0.02,61.14) | 0.95 (0.02,49.16) | 2.01 (0.03,136.00) | 4.38 (0.03,650.72) | 1.00 (0.02,48.82) | 1.29 (0.02,72.09) | 0.98 (0.02,52.39) | 1.77 (0.03,125.09) | - | 0.88 (0.02,44.60) | 4.28 (0.07,277.84) | 0.99 (0.02,49.37) | 0.38 (0,31.44) | 0.83 (0.02,42.52) | 0.72 (0.01,94.61) | 1.01 (0.02,51.66) | 1.11 (0.02,56.25) |  |
| 0.07 (0,1.18) | **ADA** | 1.42  (0.56,3.58) | 1.22 (0.55,2.68) | 0.99 (0.47,2.06) | 2.08 (0.40,10.89) | 4.53 (0.20,104.39) | 1.04 (0.53,2.03) | 1.33 (0.45,3.94) | 1.02 (0.53,1.95) | 1.83 (0.33,10.21) | - | 0.91 (0.43,1.92) | 4.43 (0.99,19.81) | 1.02 (0.61,1.73) | 0.40 (0.05,3.24) | 0.86 (0.41,1.83) | 0.75 (0.04,14.36) | 1.04 (0.47,2.28) | 1.15 (0.58,2.28) |  |
| 0.22 (0.01,4.93) | 3.39 (1.11,10.39) | **ALEFACEPT** | 0.86 (0.33,2.25) | 0.70 (0.28,1.75) | 1.47 (0.26,8.42) | 3.20 (0.13,79.25) | 0.73 (0.31,1.75) | 0.94 (0.28,3.19) | 0.72 (0.25,2.04) | 1.29 (0.20,8.21) | - | 0.64 (0.25,1.63) | 3.13 (0.60,16.20) | 0.72 (0.34,1.55) | 0.28 (0.03,2.46) | 0.61 (0.24,1.55) | 0.53 (0.03,10.68) | 0.74 (0.28,1.92) | 0.81 (0.33,1.96) |  |
| 0.13 (0.01,2.36) | 1.94 (0.99,3.82) | 0.57  (0.16,2.09) | **APRE** | 0.81 (0.37,1.77) | 1.71 (0.32,9.14) | 3.72 (0.16,88.89) | 0.85 (0.42,1.72) | 1.09 (0.36,3.36) | 0.84 (0.33,2.11) | 1.51 (0.25,8.95) | - | 0.75 (0.34,1.64) | 3.64 (0.76,17.52) | 0.84 (0.47,1.52) | 0.32 (0.04,2.71) | 0.71 (0.32,1.56) | 0.62 (0.03,11.95) | 0.86 (0.38,1.95) | 0.94 (0.45,1.96) |  |
| 0.04 (0,0.68) | 0.58 (0.37,0.92) | 0.17  (0.05,0.57) | 0.30 (0.16,0.56) | **BRODA** | 2.11 (0.40,10.99) | 4.59 (0.19,108.16) | 1.05 (0.55,2.02) | 1.35 (0.46,3.99) | 1.03 (0.43,2.48) | 1.86 (0.32,10.78) | - | 0.92 (0.44,1.92) | 4.48 (0.96,21.03) | 1.04 (0.62,1.73) | 0.40 (0.05,3.28) | 0.87 (0.43,1.79) | 0.76 (0.04,14.52) | 1.05 (0.49,2.29) | 1.16 (0.64,2.11) |  |
| 0.04 (0,1.29) | 0.60 (0.08,4.44) | 0.18  (0.02,1.74) | 0.31 (0.04,2.41) | 1.04 (0.14,7.54) | **CERTO** | 2.18 (0.07,71.39) | 0.50 (0.10,2.53) | 0.64 (0.10,4.01) | 0.49 (0.09,2.74) | 0.88 (0.09,8.79) | - | 0.44 (0.08,2.29) | 2.13 (0.25,18.10) | 0.49 (0.10,2.36) | 0.19 (0.01,2.49) | 0.41 (0.08,2.17) | 0.36 (0.01,9.79) | 0.50 (0.09,2.67) | 0.55 (0.11,2.82) |  |
| 0.25 (0.01,4.81) | 3.73 (1.79,7.76) | 1.10  (0.36,3.38) | 1.92 (0.72,5.12) | 6.38 (2.73,14.92) | 6.17 (0.74,51.08) | **CICLO** | 0.23 (0.01,5.31) | 0.29 (0.01,7.36) | 0.22 (0.01,5.41) | 0.40 (0.02,7.21) | - | 0.20 (0.01,4.75) | 0.98 (0.06,15.38) | 0.23 (0.01,5.10) | 0.09 (0,3.62) | 0.19 (0.01,4.50) | 0.17 (0,11.73) | 0.23 (0.01,5.48) | 0.25 (0.01,5.90) |  |
| 0.09 (0.01,1.58) | 1.38 (0.90,2.11) | 0.41  (0.12,1.33) | 0.71 (0.40,1.25) | 2.36 (1.83,3.04) | 2.28 (0.32,16.44) | 0.37 (0.16,0.84) | **ETA** | 1.29 (0.45,3.66) | 0.98 (0.43,2.25) | 1.77 (0.31,10.03) | - | 0.88 (0.51,1.51) | 4.28 (0.94,19.51) | 0.99 (0.65,1.51) | 0.38 (0.05,3.06) | 0.83 (0.46,1.51) | 0.72 (0.04,13.65) | 1.01 (0.53,1.89) | 1.11 (0.64,1.93) |  |
| 0.24 (0.01,4.68) | 3.64 (1.57,8.41) | 1.07  (0.27,4.19) | 1.87 (0.71,4.93) | 6.22 (2.69,14.38) | 6.01 (0.73,49.53) | 0.97 (0.33,2.85) | 2.64 (1.17,5.95) | **FUM** | 0.76 (0.23,2.51) | 1.38 (0.21,8.90) | - | 0.68 (0.23,2.04) | 3.32 (0.63,17.61) | 0.77 (0.30,1.99) | 0.30 (0.03,2.82) | 0.65 (0.22,1.93) | 0.56 (0.03,11.98) | 0.78 (0.26,2.39) | 0.86 (0.30,2.47) |  |
| 0.05 (0,0.84) | 0.71 (0.61,0.83) | 0.21  (0.07,0.64) | 0.36 (0.18,0.72) | 1.21 (0.74,1.98) | 1.17 (0.16,8.60) | 0.19 (0.09,0.40) | 0.51 (0.33,0.80) | 0.19 (0.08,0.45) | **GUSEL** | 1.80 (0.30,10.87) | - | 0.90 (0.37,2.17) | 4.35 (0.89,21.33) | 1.00 (0.49,2.04) | 0.39 (0.04,3.36) | 0.85 (0.35,2.06) | 0.74 (0.04,14.66) | 1.02 (0.41,2.57) | 1.13 (0.49,2.61) |  |
| 0.09 (0,1.67) | 1.33 (0.72,2.46) | 0.39  (0.14,1.12) | 0.69 (0.28,1.68) | 2.28 (1.07,4.85) | 2.20 (0.28,17.52) | 0.36 (0.19,0.66) | 0.96 (0.47,1.99) | 0.37 (0.14,0.99) | 1.88 (1.01,3.51) | **IFX** | - | 0.50 (0.09,2.90) | 2.41 (1.04,5.59) | 0.56 (0.10,3.00) | 0.22 (0.02,3.03) | 0.47 (0.08,2.74) | 0.41 (0.01,11.73) | 0.57 (0.10,3.37) | 0.63 (0.11,3.56) |  |
| 0.08 (0,4.35) | 1.21 (0.07,20.07) | 0.36  (0.02,7.29) | 0.62 (0.04,10.73) | 2.08 (0.13,34.17) | 2.00 (0.07,60.41) | 0.33 (0.02,5.87) | 0.88 (0.05,14.39) | 0.33 (0.02,6.00) | 1.72 (0.10,28.45) | 0.91 (0.05,16.01) | **ITO** | - | - | - | - | - | - | - | - |  |
| 0.03 (0,0.53) | 0.46 (0.29,0.73) | 0.14  (0.04,0.45) | 0.24 (0.13,0.43) | 0.78 (0.59,1.05) | 0.76 (0.10,5.52) | 0.12 (0.05,0.29) | 0.33 (0.27,0.41) | 0.13 (0.05,0.29) | 0.65 (0.39,1.07) | 0.34 (0.16,0.74) | 0.38 (0.02,6.23) | **IXE** | 4.86 (1.03,22.88) | 1.12 (0.66,1.90) | 0.43 (0.05,3.56) | 0.95 (0.46,1.94) | 0.82 (0.04,15.77) | 1.14 (0.54,2.42) | 1.26 (0.65,2.45) |  |
| 0.25 (0.01,4.69) | 3.80 (2.26,6.39) | 1.12  (0.42,3.02) | 1.96 (0.85,4.50) | 6.51 (3.29,12.87) | 6.29 (0.81,48.79) | 1.02 (0.60,1.73) | 2.76 (1.45,5.26) | 1.05 (0.41,2.67) | 5.38 (3.17,9.13) | 2.86 (2.06,3.97) | 3.14 (0.18,54.06) | 8.30 (4.18,16.50) | **MTX** | 0.23 (0.05,0.99) | 0.09 (0.01,1.09) | 0.19 (0.04,0.92) | 0.17 (0.01,4.37) | 0.24 (0.05,1.13) | 0.26 (0.06,1.19) |  |
| 0.98 (0.06,17.24) | 14.87 (10.45,21.14) | 4.39  (1.38,13.94) | 7.66 (4.30,13.66) | 25.45 (18.74,34.57) | 24.58 (3.46,174.73) | 3.99 (1.81,8.78) | 10.79 (8.47,13.73) | 4.09 (1.88,8.88) | 21.03 (14.56,30.38) | 11.18 (5.67,22.04) | 12.26 (0.76,198.53) | 32.45 (23.61,44.60) | 3.91 (2.16,7.08) | **PBO** | 0.39 (0.05,2.97) | 0.84 (0.49,1.44) | 0.73 (0.04,13.39) | 1.02 (0.57,1.83) | 1.12 (0.72,1.75) |  |
| 0.15 (0.01,3.61) | 2.25 (0.53,9.52) | 0.66  (0.11,4.08) | 1.16 (0.26,5.27) | 3.86 (0.92,16.13) | 3.73 (0.34,41.40) | 0.60 (0.12,3.01) | 1.64 (0.40,6.75) | 0.62 (0.13,3.06) | 3.19 (0.75,13.52) | 1.69 (0.36,8.01) | 1.86 (0.08,41.90) | 4.92 (1.17,20.61) | 0.59 (0.13,2.70) | 0.15 (0.04,0.61) | **PONE** | 2.18 (0.26,17.93) | 1.90 (0.05,65.94) | 2.63 (0.32,21.94) | 2.90 (0.36,23.36) |  |
| 0.04 (0,0.65) | 0.56 (0.36,0.87) | 0.17  (0.05,0.54) | 0.29 (0.16,0.52) | 0.96 (0.75,1.22) | 0.93 (0.13,6.70) | 0.15 (0.07,0.35) | 0.41 (0.33,0.50) | 0.15 (0.07,0.35) | 0.79 (0.50,1.25) | 0.42 (0.20,0.88) | 0.46 (0.03,7.57) | 1.22 (0.92,1.62) | 0.15 (0.08,0.28) | 0.04 (0.03,0.05) | 0.25 (0.06,1.03) | **SECU** | 0.87 (0.05,16.71) | 1.21 (0.56,2.61) | 1.33 (0.74,2.38) |  |
| 0.06 (0,2.01) | 0.95 (0.13,6.91) | 0.28  (0.03,2.71) | 0.49 (0.06,3.75) | 1.63 (0.23,11.74) | 1.57 (0.10,25.02) | 0.26 (0.03,2.09) | 0.69 (0.10,4.93) | 0.26 (0.03,2.14) | 1.35 (0.18,9.81) | 0.72 (0.09,5.65) | 0.78 (0.03,23.52) | 2.08 (0.29,15.00) | 0.25 (0.03,1.92) | 0.06 (0.01,0.45) | 0.42 (0.04,4.65) | 1.70 (0.24,12.18) | **TILDRA** | 1.39 (0.07,26.90) | 1.53 (0.08,28.92) |  |
| 0.12 (0.01,2.04) | 1.75 (1.09,2.82) | 0.52  (0.16,1.70) | 0.90 (0.48,1.68) | 2.99 (1.97,4.54) | 2.89 (0.40,21.07) | 0.47 (0.20,1.09) | 1.27 (0.95,1.70) | 0.48 (0.21,1.11) | 2.47 (1.54,3.96) | 1.32 (0.63,2.75) | 1.44 (0.09,23.77) | 3.82 (2.57,5.68) | 0.46 (0.24,0.89) | 0.12 (0.09,0.16) | 0.78 (0.19,3.25) | 3.12 (2.21,4.41) | 1.84 (0.25,13.27) | **TOFA** | 1.10 (0.54,2.26) |  |
| 0.05 (0,0.86) | 0.75 (0.48,1.16) | 0.22  (0.07,0.73) | 0.38 (0.21,0.70) | 1.28 (1.10,1.48) | 1.23 (0.17,8.95) | 0.20 (0.09,0.46) | 0.54 (0.44,0.67) | 0.21 (0.09,0.47) | 1.06 (0.66,1.69) | 0.56 (0.27,1.18) | 0.62 (0.04,10.11) | 1.63 (1.25,2.12) | 0.20 (0.10,0.38) | 0.05 (0.04,0.07) | 0.33 (0.08,1.38) | 1.33 (1.11,1.61) | 0.79 (0.11,5.64) | 0.43 (0.29,0.63) | **USK** |  |
| **PASI 90** | | | | | | | | | | | | | | | | | | | | |

**Appendix Table 2**. League table of probabilities $p_{i>j}$ for efficacy (PASI 90) in lower triangle and safety (serious adverse events (SAE)) in upper triangle. For both outcomes each cell contains the percentage probability that the treatment on the left produces a better outcome than the treatment on the right.

| **SAE** | | | | | | | | | | | | | | | | | | | |
| --- | --- | --- | --- | --- | --- | --- | --- | --- | --- | --- | --- | --- | --- | --- | --- | --- | --- | --- | --- |
| **ACI** | 50.7 | 43.9 | 46.8 | 50.9 | 37.3 | 28.1 | 50.0 | 45.1 | 50.3 | 39.6 | - | 52.5 | 24.7 | 50.2 | 66.6 | 53.6 | 55.2 | 49.9 | 48.0 |
| 3.3 | **ADA** | 23.1 | 31.3 | 51.4 | 19.3 | 17.3 | 46.0 | 30.2 | 47.8 | 24.4 | - | 59.6 | 2.6 | 46.6 | 80.6 | 65.1 | 57.6 | 46.0 | 34.8 |
| 17.1 | 98.4 | **ALEFACEPT** | 62.1 | 77.9 | 33.3 | 23.9 | 75.9 | 53.9 | 73.2 | 39.2 | - | 82.4 | 8.7 | 79.8 | 87.5 | 85.1 | 66.1 | 73.5 | 68.0 |
| 8.3 | 97.3 | 19.9 | **APRE** | 70.0 | 26.5 | 20.8 | 67.4 | 43.7 | 64.7 | 32.6 | - | 76.6 | 5.4 | 71.8 | 85.0 | 80.4 | 62.6 | 64.5 | 56.3 |
| 1.3 | 1.1 | 0.2 | 0.0 | **BRODA** | 18.8 | 17.2 | 44.3 | 29.4 | 47.2 | 24.5 | - | 58.5 | 2.8 | 44.6 | 80.3 | 64.5 | 57.3 | 44.7 | 31.2 |
| 3.5 | 31.1 | 6.9 | 13.2 | 51.4 | **CERTO** | 33.1 | 80.0 | 68.3 | 79.2 | 54.3 | - | 83.6 | 24.5 | 81.2 | 89.7 | 85.1 | 72.8 | 79.1 | 76.3 |
| 17.8 | 100 | 56.6 | 90.4 | 100.0 | 95.4 | **CICLO** | 82.1 | 77.2 | 82.1 | 73.1 | - | 84.0 | 50.7 | 82.5 | 90.0 | 84.8 | 79.6 | 81.8 | 80.4 |
| 5.0 | 93.1 | 6.8 | 11.9 | 100.0 | 79.3 | 0.9 | **ETA** | 31.8 | 51.6 | 25.9 | - | 67.9 | 3.0 | 52.2 | 81.8 | 72.7 | 58.5 | 49.3 | 35.9 |
| 17.3 | 99.9 | 54 | 89.8 | 100.0 | 95.2 | 48.1 | 99.0 | **FUM** | 67.1 | 36.8 | - | 75.2 | 7.9 | 70.6 | 85.5 | 78.2 | 64.4 | 66.7 | 61.0 |
| 1.9 | 0.0 | 0.3 | 0.2 | 77.7 | 56.1 | 0.0 | 0.2 | 0.0 | **GUSEL** | 26.1 | - | 59.7 | 3.5 | 49.5 | 80.5 | 64.3 | 57.9 | 48.1 | 39.0 |
| 5.3 | 81.9 | 4.0 | 20.4 | 98.3 | 77.2 | 0.1 | 46.2 | 2.3 | 97.7 | **IFX** | - | 78.2 | 2.0 | 75.2 | 87.2 | 79.9 | 69.9 | 73.3 | 70.2 |
| 10.8 | 55.4 | 25.2 | 37.3 | 69.5 | 65.5 | 22.3 | 46.4 | 22.8 | 64.7 | 47.5 | **ITO** | - | - | - | - | - | - | - | - |
| 0.8 | 0.1 | 0.1 | 0.0 | 5.2 | 39.2 | 0.0 | 0.0 | 0.0 | 4.4 | 0.3 | 24.8 | **IXE** | 2.3 | 33.4 | 78.2 | 56.1 | 55.2 | 36.4 | 25.0 |
| 17.7 | 100 | 59.0 | 94.4 | 100.0 | 96.1 | 52.9 | 99.9 | 53.7 | 100.0 | 100.0 | 78.4 | 100.0 | **MTX** | 97.6 | 97.1 | 98.1 | 85.8 | 96.5 | 95.9 |
| 49.5 | 100 | 99.4 | 100 | 100.0 | 99.9 | 100 | 100 | 100.0 | 100.0 | 100.0 | 96.1 | 100.0 | 100.0 | **PBO** | 82.0 | 73.4 | 58.3 | 47.7 | 30.8 |
| 12.1 | 86.6 | 32.9 | 57.7 | 96.8 | 85.8 | 26.9 | 75.2 | 27.9 | 94.2 | 74.7 | 65.2 | 98.5 | 25.0 | 0.4 | **PONE** | 23.5 | 36.2 | 18.5 | 15.9 |
| 1.2 | 0.5 | 0.1 | 0.0 | 36.5 | 46.9 | 0.0 | 0.0 | 0.0 | 16.0 | 1.0 | 29.4 | 91.7 | 0.0 | 0.0 | 2.7 | **SECU** | 53.7 | 31.5 | 16.7 |
| 5.9 | 48.0 | 13.6 | 24.6 | 68.6 | 62.6 | 10.2 | 35.6 | 10.5 | 61.5 | 37.5 | 44.4 | 76.5 | 9.2 | 0.3 | 24.1 | 70.1 | **TILDRA** | 41.4 | 38.8 |
| 7.0 | 98.9 | 13.8 | 37.2 | 100.0 | 85.3 | 4.0 | 94.5 | 4.2 | 100.0 | 76.7 | 60.1 | 100.0 | 1.0 | 0.0 | 36.4 | 100 | 72.7 | **TOFA** | 39.6 |
| 2.0 | 9.7 | 0.7 | 0.1 | 99.9 | 58.3 | 0.0 | 0.0 | 0.0 | 59.1 | 6.4 | 36.7 | 100.0 | 0.0 | 0.0 | 6.4 | 99.9 | 40.5 | 0.0 | **USK** |
| **PASI 90** | | | | | | | | | | | | | | | | | | | |

**Appendix Table 3**. Confidence in the evidence for efficacy (PASI 90) as obtained by CINeMA.

| **Comparison** | **Number of studies** | **Within-study bias^1^** | **Across-studies bias^2^** | **Indirectness^1^** | **Imprecision^3^** | **Heterogeneity^3^** | **Incoherence^4^** | **Confidence rating^5^** | **Probability that the evidence is trustworthy^5^** |
| --- | --- | --- | --- | --- | --- | --- | --- | --- | --- |
| ***Mixed evidence*** |  |  |  |  |  |  |  |  |  |
| **ACI:ETA** | 1 | Major concerns | Undetected | No concerns | Some concerns | No concerns | No concerns | Low | 0.3 |
| **ADA:GUSEL** | 3 | No concerns | Undetected | No concerns | No concerns | No concerns | Some concerns | Moderate | 0.7 |
| **ADA:MTX** | 1 | No concerns | Undetected | No concerns | No concerns | No concerns | Some concerns | Moderate | 0.7 |
| **ADA:PBO** | 8 | No concerns | Undetected | No concerns | No concerns | No concerns | No concerns | High | 0.9 |
| **ALEFACEPT:MTX** | 1 | Major concerns | Undetected | No concerns | Major concerns | No concerns | No concerns | Very low | 0.1 |
| **APRE:ETA** | 1 | Some concerns | Undetected | No concerns | Some concerns | No concerns | No concerns | Moderate | 0.7 |
| **APRE:PBO** | 4 | Some concerns | Undetected | Some concerns | No concerns | No concerns | No concerns | Moderate | 0.7 |
| **BRODA:PBO** | 5 | Some concerns | Undetected | No concerns | No concerns | No concerns | No concerns | Moderate | 0.7 |
| **BRODA:USK** | 2 | Some concerns | Undetected | No concerns | No concerns | No concerns | Some concerns | Moderate | 0.7 |
| **CERTO:PBO** | 1 | No concerns | Undetected | Some concerns | No concerns | No concerns | No concerns | Moderate | 0.7 |
| **CICLO:MTX** | 2 | Major concerns | Undetected | No concerns | No concerns | No concerns | No concerns | Moderate | 0.7 |
| **ETA:IXE** | 2 | No concerns | Undetected | No concerns | No concerns | No concerns | Some concerns | Moderate | 0.7 |
| **ETA:PBO** | 12 | No concerns | Undetected | No concerns | No concerns | No concerns | No concerns | High | 0.9 |
| **ETA:SECU** | 1 | No concerns | Undetected | No concerns | Some concerns | No concerns | No concerns | Moderate | 0.7 |
| **ETA:TOFA** | 1 | No concerns | Undetected | No concerns | No concerns | No concerns | Some concerns | Moderate | 0.7 |
| **ETA:USK** | 1 | Some concerns | Undetected | No concerns | Some concerns | No concerns | No concerns | Moderate | 0.7 |
| **FUM:MTX** | 1 | Some concerns | Undetected | No concerns | Major concerns | No concerns | No concerns | Moderate | 0.7 |
| **FUM:PBO** | 1 | Major concerns | Undetected | Some concerns | Some concerns | No concerns | Some concerns | Very low | 0.1 |
| **GUSEL:PBO** | 3 | No concerns | Undetected | No concerns | No concerns | No concerns | No concerns | High | 0.9 |
| **IFX:MTX** | 1 | Major concerns | Undetected | No concerns | No concerns | Some concerns | No concerns | Moderate | 0.7 |
| **ITO:PBO** | 1 | Some concerns | Undetected | Some concerns | Some concerns | No concerns | No concerns | Low | 0.3 |
| **IXE:PBO** | 4 | No concerns | Undetected | No concerns | No concerns | No concerns | No concerns | High | 0.9 |
| **MTX:PBO** | 2 | No concerns | Undetected | No concerns | No concerns | No concerns | Some concerns | Moderate | 0.7 |
| **PBO:PONE** | 1 | No concerns | Undetected | Some concerns | Some concerns | No concerns | No concerns | Moderate | 0.7 |
| **PBO:SECU** | 7 | No concerns | Undetected | No concerns | No concerns | No concerns | No concerns | High | 0.9 |
| **PBO:TILDRA** | 1 | Some concerns | Undetected | Some concerns | No concerns | No concerns | No concerns | Moderate | 0.7 |
| **PBO:TOFA** | 4 | Some concerns | Undetected | Some concerns | No concerns | No concerns | No concerns | Moderate | 0.7 |
| **PBO:USK** | 7 | Some concerns | Undetected | No concerns | No concerns | No concerns | No concerns | Moderate | 0.7 |
| **SECU:USK** | 1 | No concerns | Undetected | No concerns | No concerns | No concerns | No concerns | High | 0.9 |
| ***Indirect Evidence*** |  |  |  |  |  |  |  |  |  |
| **ACI:ADA** | 0 | Some concerns | Undetected | No concerns | Some concerns | No concerns | Some concerns | Low | 0.3 |
| **ACI:ALEFACEPT** | 0 | Some concerns | Undetected | No concerns | Major concerns | No concerns | Some concerns | Low | 0.3 |
| **ACI:APRE** | 0 | Some concerns | Undetected | No concerns | Major concerns | No concerns | Some concerns | Low | 0.3 |
| **ACI:BRODA** | 0 | Some concerns | Undetected | No concerns | Some concerns | No concerns | Some concerns | Low | 0.3 |
| **ACI:CERTO** | 0 | Some concerns | Undetected | No concerns | Some concerns | No concerns | Some concerns | Low | 0.3 |
| **ACI:CICLO** | 0 | Some concerns | Undetected | No concerns | Major concerns | No concerns | Some concerns | Low | 0.3 |
| **ACI:FUM** | 0 | Some concerns | Undetected | No concerns | Major concerns | No concerns | Some concerns | Low | 0.3 |
| **ACI:GUSEL** | 0 | Some concerns | Undetected | No concerns | Some concerns | No concerns | Some concerns | Low | 0.3 |
| **ACI:IFX** | 0 | Some concerns | Undetected | No concerns | Some concerns | No concerns | Some concerns | Low | 0.3 |
| **ACI:ITO** | 0 | Some concerns | Undetected | No concerns | Major concerns | No concerns | Some concerns | Low | 0.3 |
| **ACI:IXE** | 0 | Some concerns | Undetected | No concerns | Some concerns | No concerns | Some concerns | Low | 0.3 |
| **ACI:MTX** | 0 | Some concerns | Undetected | No concerns | Major concerns | No concerns | Some concerns | Low | 0.3 |
| **ACI:PBO** | 0 | Some concerns | Undetected | No concerns | Major concerns | No concerns | Some concerns | Low | 0.3 |
| **ACI:PONE** | 0 | Some concerns | Undetected | No concerns | Major concerns | No concerns | Some concerns | Low | 0.3 |
| **ACI:SECU** | 0 | Some concerns | Undetected | No concerns | Some concerns | No concerns | Some concerns | Low | 0.3 |
| **ACI:TILDRA** | 0 | Some concerns | Undetected | No concerns | Major concerns | No concerns | Some concerns | Low | 0.3 |
| **ACI:TOFA** | 0 | Some concerns | Undetected | No concerns | Major concerns | No concerns | Some concerns | Low | 0.3 |
| **ACI:USK** | 0 | Some concerns | Undetected | No concerns | Some concerns | No concerns | Some concerns | Low | 0.3 |
| **ADA:ALEFACEPT** | 0 | Some concerns | Undetected | No concerns | Some concerns | No concerns | Some concerns | Low | 0.3 |
| **ADA:APRE** | 0 | No concerns | Undetected | No concerns | Some concerns | No concerns | Some concerns | Moderate | 0.7 |
| **ADA:BRODA** | 0 | No concerns | Undetected | No concerns | Some concerns | No concerns | Some concerns | Moderate | 0.7 |
| **ADA:CERTO** | 0 | No concerns | Undetected | Some concerns | Major concerns | No concerns | Some concerns | Low | 0.3 |
| **ADA:CICLO** | 0 | Some concerns | Undetected | No concerns | Some concerns | No concerns | Some concerns | Low | 0.3 |
| **ADA:ETA** | 0 | No concerns | Undetected | No concerns | Some concerns | No concerns | Some concerns | Moderate | 0.7 |
| **ADA:FUM** | 0 | Some concerns | Undetected | Some concerns | Some concerns | No concerns | Some concerns | Very low | 0.1 |
| **ADA:IFX** | 0 | Some concerns | Undetected | No concerns | Some concerns | No concerns | Some concerns | Low | 0.3 |
| **ADA:ITO** | 0 | Some concerns | Undetected | Some concerns | Major concerns | No concerns | Some concerns | Very low | 0.1 |
| **ADA:IXE** | 0 | No concerns | Undetected | No concerns | Some concerns | No concerns | Some concerns | Moderate | 0.7 |
| **ADA:PONE** | 0 | No concerns | Undetected | Some concerns | Some concerns | No concerns | Some concerns | Low | 0.3 |
| **ADA:SECU** | 0 | No concerns | Undetected | No concerns | Some concerns | No concerns | Some concerns | Moderate | 0.7 |
| **ADA:TILDRA** | 0 | Some concerns | Undetected | Some concerns | Major concerns | No concerns | Some concerns | Very low | 0.1 |
| **ADA:TOFA** | 0 | No concerns | Undetected | No concerns | Some concerns | No concerns | Some concerns | Moderate | 0.7 |
| **ADA:USK** | 0 | No concerns | Undetected | No concerns | Some concerns | No concerns | Some concerns | Moderate | 0.7 |
| **ALEFACEPT:APRE** | 0 | Some concerns | Undetected | No concerns | Major concerns | No concerns | Some concerns | Low | 0.3 |
| **ALEFACEPT:BRODA** | 0 | Some concerns | Undetected | No concerns | Some concerns | No concerns | Some concerns | Low | 0.3 |
| **ALEFACEPT:CERTO** | 0 | Some concerns | Undetected | No concerns | Some concerns | No concerns | Some concerns | Low | 0.3 |
| **ALEFACEPT:CICLO** | 0 | Major concerns | Undetected | No concerns | Major concerns | No concerns | Some concerns | Very low | 0.1 |
| **ALEFACEPT:ETA** | 0 | Some concerns | Undetected | No concerns | Some concerns | No concerns | Some concerns | Low | 0.3 |
| **ALEFACEPT:FUM** | 0 | Some concerns | Undetected | No concerns | Major concerns | No concerns | Some concerns | Low | 0.3 |
| **ALEFACEPT:GUSEL** | 0 | Some concerns | Undetected | No concerns | Some concerns | No concerns | Some concerns | Low | 0.3 |
| **ALEFACEPT:IFX** | 0 | Major concerns | Undetected | No concerns | Some concerns | No concerns | Some concerns | Low | 0.3 |
| **ALEFACEPT:ITO** | 0 | Some concerns | Undetected | No concerns | Major concerns | No concerns | Some concerns | Low | 0.3 |
| **ALEFACEPT:IXE** | 0 | Some concerns | Undetected | No concerns | No concerns | No concerns | Some concerns | Moderate | 0.7 |
| **ALEFACEPT:PBO** | 0 | Some concerns | Undetected | No concerns | Some concerns | No concerns | Some concerns | Low | 0.3 |
| **ALEFACEPT:PONE** | 0 | Some concerns | Undetected | No concerns | Major concerns | No concerns | Some concerns | Low | 0.3 |
| **ALEFACEPT:SECU** | 0 | Some concerns | Undetected | No concerns | Some concerns | No concerns | Some concerns | Low | 0.3 |
| **ALEFACEPT:TILDRA** | 0 | Some concerns | Undetected | No concerns | Major concerns | No concerns | Some concerns | Low | 0.3 |
| **ALEFACEPT:TOFA** | 0 | Some concerns | Undetected | No concerns | Some concerns | No concerns | Some concerns | Low | 0.3 |
| **ALEFACEPT:USK** | 0 | Some concerns | Undetected | No concerns | Some concerns | No concerns | Some concerns | Low | 0.3 |
| **APRE:BRODA** | 0 | Some concerns | Undetected | No concerns | Some concerns | No concerns | Some concerns | Low | 0.3 |
| **APRE:CERTO** | 0 | No concerns | Undetected | Some concerns | Major concerns | No concerns | Some concerns | Low | 0.3 |
| **APRE:CICLO** | 0 | Some concerns | Undetected | No concerns | Some concerns | No concerns | Some concerns | Low | 0.3 |
| **APRE:FUM** | 0 | Some concerns | Undetected | Some concerns | Some concerns | No concerns | Some concerns | Very low | 0.1 |
| **APRE:GUSEL** | 0 | No concerns | Undetected | No concerns | Some concerns | No concerns | Some concerns | Moderate | 0.7 |
| **APRE:IFX** | 0 | Some concerns | Undetected | No concerns | Some concerns | No concerns | Some concerns | Low | 0.3 |
| **APRE:ITO** | 0 | Some concerns | Undetected | Some concerns | Major concerns | No concerns | Some concerns | Very low | 0.1 |
| **APRE:IXE** | 0 | No concerns | Undetected | No concerns | No concerns | No concerns | Some concerns | Moderate | 0.7 |
| **APRE:MTX** | 0 | No concerns | Undetected | No concerns | Some concerns | No concerns | Some concerns | Moderate | 0.7 |
| **APRE:PONE** | 0 | No concerns | Undetected | Some concerns | Major concerns | No concerns | Some concerns | Low | 0.3 |
| **APRE:SECU** | 0 | Some concerns | Undetected | No concerns | Some concerns | No concerns | Some concerns | Low | 0.3 |
| **APRE:TILDRA** | 0 | Some concerns | Undetected | Some concerns | Major concerns | No concerns | Some concerns | Very low | 0.1 |
| **APRE:TOFA** | 0 | Some concerns | Undetected | No concerns | Some concerns | No concerns | Some concerns | Low | 0.3 |
| **APRE:USK** | 0 | Some concerns | Undetected | No concerns | Some concerns | No concerns | Some concerns | Low | 0.3 |
| **BRODA:CERTO** | 0 | No concerns | Undetected | Some concerns | Major concerns | No concerns | Some concerns | Low | 0.3 |
| **BRODA:CICLO** | 0 | Some concerns | Undetected | No concerns | No concerns | No concerns | Some concerns | Moderate | 0.7 |
| **BRODA:ETA** | 0 | Some concerns | Undetected | No concerns | Some concerns | No concerns | Some concerns | Low | 0.3 |
| **BRODA:FUM** | 0 | Some concerns | Undetected | No concerns | No concerns | No concerns | Some concerns | Moderate | 0.7 |
| **BRODA:GUSEL** | 0 | No concerns | Undetected | No concerns | No concerns | Some concerns | Some concerns | Moderate | 0.7 |
| **BRODA:IFX** | 0 | Some concerns | Undetected | No concerns | Some concerns | No concerns | Some concerns | Low | 0.3 |
| **BRODA:ITO** | 0 | Some concerns | Undetected | Some concerns | Major concerns | No concerns | Some concerns | Very low | 0.1 |
| **BRODA:IXE** | 0 | Some concerns | Undetected | No concerns | No concerns | No concerns | Some concerns | Moderate | 0.7 |
| **BRODA:MTX** | 0 | No concerns | Undetected | No concerns | No concerns | No concerns | Some concerns | Moderate | 0.7 |
| **BRODA:PONE** | 0 | No concerns | Undetected | Some concerns | Some concerns | No concerns | Some concerns | Low | 0.3 |
| **BRODA:SECU** | 0 | Some concerns | Undetected | No concerns | No concerns | No concerns | Some concerns | Moderate | 0.7 |
| **BRODA:TILDRA** | 0 | Some concerns | Undetected | Some concerns | Major concerns | No concerns | Some concerns | Very low | 0.1 |
| **BRODA:TOFA** | 0 | Some concerns | Undetected | No concerns | No concerns | No concerns | Some concerns | Moderate | 0.7 |
| **CERTO:CICLO** | 0 | Some concerns | Undetected | No concerns | Some concerns | No concerns | Some concerns | Low | 0.3 |
| **CERTO:ETA** | 0 | No concerns | Undetected | Some concerns | Major concerns | No concerns | Some concerns | Low | 0.3 |
| **CERTO:FUM** | 0 | Some concerns | Undetected | Some concerns | Some concerns | No concerns | Some concerns | Very low | 0.1 |
| **CERTO:GUSEL** | 0 | No concerns | Undetected | Some concerns | Major concerns | No concerns | Some concerns | Low | 0.3 |
| **CERTO:IFX** | 0 | Some concerns | Undetected | No concerns | Major concerns | No concerns | Some concerns | Low | 0.3 |
| **CERTO:ITO** | 0 | Some concerns | Undetected | Some concerns | Major concerns | No concerns | Some concerns | Low | 0.3 |
| **CERTO:IXE** | 0 | No concerns | Undetected | Some concerns | Major concerns | No concerns | Some concerns | Low | 0.3 |
| **CERTO:MTX** | 0 | No concerns | Undetected | Some concerns | Some concerns | No concerns | Some concerns | Low | 0.3 |
| **CERTO:PONE** | 0 | No concerns | Undetected | Some concerns | Major concerns | No concerns | Some concerns | Low | 0.3 |
| **CERTO:SECU** | 0 | No concerns | Undetected | Some concerns | Major concerns | No concerns | Some concerns | Low | 0.3 |
| **CERTO:TILDRA** | 0 | Some concerns | Undetected | Some concerns | Major concerns | No concerns | Some concerns | Very low | 0.1 |
| **CERTO:TOFA** | 0 | No concerns | Undetected | Some concerns | Major concerns | No concerns | Some concerns | Low | 0.3 |
| **CERTO:USK** | 0 | No concerns | Undetected | Some concerns | Major concerns | No concerns | Some concerns | Low | 0.3 |
| **CICLO:ETA** | 0 | Some concerns | Undetected | No concerns | Some concerns | No concerns | Some concerns | Low | 0.3 |
| **CICLO:FUM** | 0 | Some concerns | Undetected | No concerns | Major concerns | No concerns | Some concerns | Low | 0.3 |
| **CICLO:GUSEL** | 0 | Some concerns | Undetected | No concerns | No concerns | No concerns | Some concerns | Moderate | 0.7 |
| **CICLO:IFX** | 0 | Major concerns | Undetected | No concerns | Some concerns | No concerns | Some concerns | Low | 0.3 |
| **CICLO:ITO** | 0 | Some concerns | Undetected | No concerns | Major concerns | No concerns | Some concerns | Low | 0.3 |
| **CICLO:IXE** | 0 | Some concerns | Undetected | No concerns | No concerns | No concerns | Some concerns | Moderate | 0.7 |
| **CICLO:PBO** | 0 | Some concerns | Undetected | No concerns | Some concerns | No concerns | Some concerns | Low | 0.3 |
| **CICLO:PONE** | 0 | Some concerns | Undetected | No concerns | Major concerns | No concerns | Some concerns | Low | 0.3 |
| **CICLO:SECU** | 0 | Some concerns | Undetected | No concerns | No concerns | No concerns | Some concerns | Moderate | 0.7 |
| **CICLO:TILDRA** | 0 | Some concerns | Undetected | No concerns | Major concerns | No concerns | Some concerns | Low | 0.3 |
| **CICLO:TOFA** | 0 | Some concerns | Undetected | No concerns | Some concerns | No concerns | Some concerns | Low | 0.3 |
| **CICLO:USK** | 0 | Some concerns | Undetected | No concerns | No concerns | No concerns | Some concerns | Moderate | 0.7 |
| **ETA:FUM** | 0 | Some concerns | Undetected | Some concerns | Some concerns | No concerns | Some concerns | Very low | 0.1 |
| **ETA:GUSEL** | 0 | No concerns | Undetected | No concerns | Some concerns | No concerns | Some concerns | Moderate | 0.7 |
| **ETA:IFX** | 0 | Some concerns | Undetected | No concerns | Major concerns | No concerns | Some concerns | Low | 0.3 |
| **ETA:ITO** | 0 | Some concerns | Undetected | Some concerns | Major concerns | No concerns | Some concerns | Very low | 0.1 |
| **ETA:MTX** | 0 | No concerns | Undetected | No concerns | Some concerns | No concerns | Some concerns | Moderate | 0.7 |
| **ETA:PONE** | 0 | No concerns | Undetected | Some concerns | Major concerns | No concerns | Some concerns | Low | 0.3 |
| **ETA:TILDRA** | 0 | Some concerns | Undetected | Some concerns | Major concerns | No concerns | Some concerns | Very low | 0.1 |
| **FUM:GUSEL** | 0 | Some concerns | Undetected | No concerns | No concerns | No concerns | Some concerns | Moderate | 0.7 |
| **FUM:IFX** | 0 | Some concerns | Undetected | No concerns | Some concerns | No concerns | Some concerns | Low | 0.3 |
| **FUM:ITO** | 0 | Some concerns | Undetected | Some concerns | Major concerns | No concerns | Some concerns | Very low | 0.1 |
| **FUM:IXE** | 0 | Some concerns | Undetected | No concerns | No concerns | No concerns | Some concerns | Moderate | 0.7 |
| **FUM:PONE** | 0 | Some concerns | Undetected | Some concerns | Major concerns | No concerns | Some concerns | Very low | 0.1 |
| **FUM:SECU** | 0 | Some concerns | Undetected | Some concerns | No concerns | No concerns | Some concerns | Low | 0.3 |
| **FUM:TILDRA** | 0 | Some concerns | Undetected | Some concerns | Major concerns | No concerns | Some concerns | Very low | 0.1 |
| **FUM:TOFA** | 0 | Some concerns | Undetected | Some concerns | Some concerns | No concerns | Some concerns | Very low | 0.1 |
| **FUM:USK** | 0 | Some concerns | Undetected | Some concerns | No concerns | No concerns | Some concerns | Low | 0.3 |
| **GUSEL:IFX** | 0 | Some concerns | Undetected | No concerns | Some concerns | No concerns | Some concerns | Low | 0.3 |
| **GUSEL:ITO** | 0 | No concerns | Undetected | Some concerns | Major concerns | No concerns | Some concerns | Low | 0.3 |
| **GUSEL:IXE** | 0 | No concerns | Undetected | No concerns | Some concerns | No concerns | Some concerns | Moderate | 0.7 |
| **GUSEL:MTX** | 0 | No concerns | Undetected | No concerns | No concerns | No concerns | Some concerns | Moderate | 0.7 |
| **GUSEL:PONE** | 0 | No concerns | Undetected | Some concerns | Some concerns | No concerns | Some concerns | Low | 0.3 |
| **GUSEL:SECU** | 0 | No concerns | Undetected | No concerns | No concerns | Some concerns | Some concerns | Moderate | 0.7 |
| **GUSEL:TILDRA** | 0 | No concerns | Undetected | Some concerns | Major concerns | No concerns | Some concerns | Low | 0.3 |
| **GUSEL:TOFA** | 0 | No concerns | Undetected | No concerns | Some concerns | No concerns | Some concerns | Moderate | 0.7 |
| **GUSEL:USK** | 0 | No concerns | Undetected | No concerns | No concerns | No concerns | Some concerns | Moderate | 0.7 |
| **IFX:ITO** | 0 | Some concerns | Undetected | No concerns | Major concerns | No concerns | Some concerns | Low | 0.3 |
| **IFX:IXE** | 0 | Some concerns | Undetected | No concerns | Some concerns | No concerns | Some concerns | Low | 0.3 |
| **IFX:PBO** | 0 | Some concerns | Undetected | No concerns | No concerns | No concerns | Some concerns | Moderate | 0.7 |
| **IFX:PONE** | 0 | Some concerns | Undetected | No concerns | Major concerns | No concerns | Some concerns | Low | 0.3 |
| **IFX:SECU** | 0 | Some concerns | Undetected | No concerns | Some concerns | No concerns | Some concerns | Low | 0.3 |
| **IFX:TILDRA** | 0 | Some concerns | Undetected | No concerns | Major concerns | No concerns | Some concerns | Low | 0.3 |
| **IFX:TOFA** | 0 | Some concerns | Undetected | No concerns | Some concerns | No concerns | Some concerns | Low | 0.3 |
| **IFX:USK** | 0 | Some concerns | Undetected | No concerns | Some concerns | No concerns | Some concerns | Low | 0.3 |
| **ITO:IXE** | 0 | Some concerns | Undetected | Some concerns | Major concerns | No concerns | Some concerns | Very low | 0.1 |
| **ITO:MTX** | 0 | No concerns | Undetected | Some concerns | Major concerns | No concerns | Some concerns | Low | 0.3 |
| **ITO:PONE** | 0 | Some concerns | Undetected | Some concerns | Major concerns | No concerns | Some concerns | Very low | 0.1 |
| **ITO:SECU** | 0 | Some concerns | Undetected | Some concerns | Major concerns | No concerns | Some concerns | Very low | 0.1 |
| **ITO:TILDRA** | 0 | Some concerns | Undetected | Some concerns | Major concerns | No concerns | Some concerns | Very low | 0.1 |
| **ITO:TOFA** | 0 | Some concerns | Undetected | Some concerns | Major concerns | No concerns | Some concerns | Very low | 0.1 |
| **ITO:USK** | 0 | Some concerns | Undetected | Some concerns | Major concerns | No concerns | Some concerns | Very low | 0.1 |
| **IXE:MTX** | 0 | No concerns | Undetected | No concerns | No concerns | No concerns | Some concerns | Moderate | 0.7 |
| **IXE:PONE** | 0 | No concerns | Undetected | Some concerns | Some concerns | No concerns | Some concerns | Low | 0.3 |
| **IXE:SECU** | 0 | No concerns | Undetected | No concerns | No concerns | No concerns | Some concerns | Moderate | 0.7 |
| **IXE:TILDRA** | 0 | Some concerns | Undetected | Some concerns | Major concerns | No concerns | Some concerns | Very low | 0.1 |
| **IXE:TOFA** | 0 | No concerns | Undetected | No concerns | No concerns | No concerns | Some concerns | Moderate | 0.7 |
| **IXE:USK** | 0 | Some concerns | Undetected | No concerns | Some concerns | No concerns | Some concerns | Low | 0.3 |
| **MTX:PONE** | 0 | No concerns | Undetected | Some concerns | Major concerns | No concerns | Some concerns | Low | 0.3 |
| **MTX:SECU** | 0 | No concerns | Undetected | No concerns | No concerns | No concerns | Some concerns | Moderate | 0.7 |
| **MTX:TILDRA** | 0 | No concerns | Undetected | Some concerns | Some concerns | Some concerns | Some concerns | Very low | 0.1 |
| **MTX:TOFA** | 0 | No concerns | Undetected | No concerns | Some concerns | No concerns | Some concerns | Moderate | 0.7 |
| **MTX:USK** | 0 | No concerns | Undetected | No concerns | No concerns | No concerns | Some concerns | Moderate | 0.7 |
| **PONE:SECU** | 0 | No concerns | Undetected | Some concerns | Some concerns | No concerns | Some concerns | Low | 0.3 |
| **PONE:TILDRA** | 0 | Some concerns | Undetected | Some concerns | Major concerns | No concerns | Some concerns | Very low | 0.1 |
| **PONE:TOFA** | 0 | No concerns | Undetected | Some concerns | Major concerns | No concerns | Some concerns | Low | 0.3 |
| **PONE:USK** | 0 | No concerns | Undetected | Some concerns | Some concerns | No concerns | Some concerns | Low | 0.3 |
| **SECU:TILDRA** | 0 | Some concerns | Undetected | Some concerns | Major concerns | No concerns | Some concerns | Very low | 0.1 |
| **SECU:TOFA** | 0 | No concerns | Undetected | No concerns | No concerns | No concerns | Some concerns | Moderate | 0.7 |
| **TILDRA:TOFA** | 0 | Some concerns | Undetected | Some concerns | Major concerns | No concerns | Some concerns | Very low | 0.1 |
| **TILDRA:USK** | 0 | Some concerns | Undetected | Some concerns | Major concerns | No concerns | Some concerns | Very low | 0.1 |
| **TOFA:USK** | 0 | Some concerns | Undetected | No concerns | Some concerns | No concerns | Some concerns | Low | 0.3 |
| ^1^ Each network meta-analysis estimate (mixed or indirect) is a weighted linear combination of the relative effects of the available studies in the network. So, each study is first evaluated in terms of its risk of bias (for blinding, allocation concealment, etc.) and its indirectness to the research question and then these evaluations are combined with their contributions (weights) to the network estimates to obtain the confidence for the two domains for every comparison.  ^2^ The judgment for this domain was based on the fact that all Cochrane reviews follow a comprehensive search strategy including search for unpublished studies. Also the comparison-adjusted funnel plots appeared generally symmetrical.  ^3^ An odds ratio of two was considered a clinically important difference in this network which results in the range of clinical equivalence (0.5,2) between two drugs. The judgments for imprecision are based on whether the confidence interval of each network estimate (mixed or indirect) includes values in this interval. For heterogeneity the judgments consider whether prediction intervals (i.e. the interval within which the effect of future study is expected to lie) include or exclude values in this interval when the confidence intervals do so.  ^4^ The judgments are based on the results of the side-splitting method for incoherence and the design-by-treatment interaction model (or only the latter for indirect comparisons)  ^5^ Based on the domain-specific judgments we rate each comparison as being of high, moderate, low or very low confidence and we translate these ratings into $P(trust)=0.9,0.7,0.3,0.1$ respectively. | | | | | | | | | |

**Appendix Table 4**. Probabilities of trusting to use each treatment based on the confidence of the evidence in the respective pairwise comparison.

| **ACI** | 0.30 | 0.30 | 0.30 | 0.30 | 0.30 | 0.30 | 0.30 | 0.30 | 0.30 | 0.30 | 0.30 | 0.30 | 0.30 | 0.15 | 0.30 | 0.30 | 0.30 | 0.30 | 0.30 |
| --- | --- | --- | --- | --- | --- | --- | --- | --- | --- | --- | --- | --- | --- | --- | --- | --- | --- | --- | --- |
| 0 | **ADA** | 0 | 0.35 | 0.35 | 0.15 | 0 | 0.35 | 0 | 0.35 | 0.15 | 0.05 | 0.70 | 0 | 0 | 0 | 0.35 | 0.05 | 0.35 | 0.35 |
| 0 | 0.30 | **ALEFACEPT** | 0.15 | 0.30 | 0.30 | 0.05 | 0.30 | 0.15 | 0.30 | 0.30 | 0.30 | 0.70 | 0.05 | 0 | 0.15 | 0.30 | 0.30 | 0.15 | 0.30 |
| 0 | 0.35 | 0.15 | **APRE** | 0.30 | 0.30 | 0.15 | 0.35 | 0.05 | 0.70 | 0.15 | 0.05 | 0.70 | 0.35 | 0 | 0.15 | 0.30 | 0.10 | 0.15 | 0.30 |
| 0 | 0.35 | 0 | 0 | **BRODA** | 0.15 | 0 | 0 | 0 | 0.35 | 0 | 0 | 0.35 | 0 | 0 | 0 | 0.35 | 0.05 | 0 | 0.35 |
| 0 | 0.15 | 0 | 0 | 0.15 | **CERTO** | 0 | 0 | 0 | 0.15 | 0 | 0 | 0.15 | 0 | 0 | 0 | 0.15 | 0.05 | 0 | 0.15 |
| 0 | 0.30 | 0.05 | 0.15 | 0.70 | 0.30 | **CICLO** | 0.30 | 0.15 | 0.70 | 0.30 | 0.30 | 0.70 | 0.35 | 0 | 0.15 | 0.70 | 0.30 | 0.30 | 0.70 |
| 0 | 0.35 | 0 | 0.35 | 0.30 | 0.30 | 0 | **ETA** | 0 | 0.35 | 0.15 | 0.05 | 0.70 | 0 | 0 | 0.15 | 0.70 | 0.05 | 0.35 | 0.35 |
| 0 | 0.10 | 0.15 | 0.05 | 0.70 | 0.10 | 0.15 | 0.10 | **FUM** | 0.70 | 0.30 | 0.10 | 0.70 | 0.35 | 0 | 0.05 | 0.30 | 0.10 | 0.10 | 0.30 |
| 0 | 0.35 | 0 | 0 | 0.35 | 0.15 | 0 | 0.35 | 0 | **GUSEL** | 0.15 | 0.15 | 0.35 | 0 | 0 | 0 | 0.35 | 0.15 | 0 | 0.35 |
| 0 | 0.15 | 0 | 0.15 | 0.30 | 0.30 | 0 | 0.15 | 0 | 0.15 | **IFX** | 0.15 | 0.30 | 0 | 0 | 0.15 | 0.30 | 0.15 | 0.15 | 0.15 |
| 0 | 0.05 | 0 | 0.05 | 0.10 | 0.30 | 0 | 0.05 | 0 | 0.15 | 0.15 | **ITO** | 0.10 | 0 | 0 | 0.05 | 0.10 | 0.05 | 0.05 | 0.05 |
| 0 | 0 | 0 | 0 | 0.35 | 0.15 | 0 | 0 | 0 | 0.35 | 0 | 0 | **IXE** | 0 | 0 | 0 | 0.35 | 0 | 0 | 0.15 |
| 0 | 0.70 | 0.05 | 0.35 | 0.70 | 0.30 | 0.35 | 0.70 | 0.35 | 0.70 | 0.70 | 0.30 | 0.70 | **MTX** | 0 | 0.15 | 0.70 | 0.10 | 0.70 | 0.70 |
| 0.15 | 0.90 | 0.30 | 0 | 0.70 | 0.70 | 0.30 | 0.90 | 0.10 | 0.90 | 0.70 | 0.30 | 0.90 | 0.70 | **PBO** | 0.70 | 0.90 | 0.70 | 0.70 | 0.70 |
| 0 | 0.30 | 0.15 | 0.15 | 0.30 | 0.30 | 0.15 | 0.15 | 0.05 | 0.30 | 0.15 | 0.05 | 0.30 | 0.15 | 0 | **PONE** | 0.30 | 0.10 | 0.15 | 0.30 |
| 0 | 0.35 | 0 | 0 | 0.35 | 0.15 | 0 | 0 | 0 | 0.35 | 0 | 0 | 0.35 | 0 | 0 | 0 | **SECU** | 0.05 | 0 | 0.45 |
| 0 | 0.05 | 0 | 0 | 0.05 | 0.05 | 0 | 0.05 | 0 | 0.15 | 0.15 | 0.05 | 0.10 | 0 | 0 | 0 | 0.05 | **TILDRA** | 0.05 | 0.05 |
| 0 | 0.35 | 0.15 | 0.15 | 0.70 | 0.30 | 0 | 0.35 | 0 | 0.70 | 0.15 | 0.05 | 0.70 | 0 | 0 | 0.15 | 0.70 | 0.05 | **TOFA** | 0.30 |
| 0 | 0.35 | 0 | 0 | 0.35 | 0.15 | 0 | 0.35 | 0 | 0.35 | 0.15 | 0.05 | 0.15 | 0 | 0 | 0 | 0.45 | 0.05 | 0 | **USK** |

**Appendix Table 5**. Information on treatment ranking for the psoriasis network in terms of treatment cost. Grey cells correspond to the five top positions.

| **Drug** | **Average cost per person per year in France** | $\boldsymbol{c}_{\boldsymbol{i}}$ | $\boldsymbol{l}_{\boldsymbol{i}}^{\boldsymbol{(0)}}$ | **cost rank** |
| --- | --- | --- | --- | --- |
| ACI | <2000 | ***0.879*** | ***0.162*** | ***1*** |
| ADA | 12000 | 0.273 | 0.05 | 8 |
| ALEFACEPT | not commercialised | - | 0 | 10 |
| APRE | 7000 | ***0.576*** | ***0.106*** | ***5*** |
| BRODA | 15000-18000 | 0 | 0 | 10 |
| CERTO | not commercialised | - | 0 | 10 |
| CICLO | <2000 | ***0.879*** | ***0.162*** | ***1*** |
| ETA | 10000 | 0.394 | 0.073 | 6 |
| FUM | <2000 | ***0.879*** | ***0.162*** | ***1*** |
| GUSEL | not commercialised | - | 0 | 10 |
| IFX | 10000 | 0.394 | 0.073 | 6 |
| ITO | not commercialised | - | 0 | 10 |
| IXE | 15000-18000 | 0 | 0 | 10 |
| MTX | <2000 | ***0.879*** | ***0.162*** | ***1*** |
| PBO | not commercialised | - | 0 | 10 |
| PONE | not commercialised | - | 0 | 10 |
| SECU | 15000-18000 | 0 | 0 | 10 |
| TILDRA | not commercialised | - | 0 | 10 |
| TOFA | not commercialised | - | 0 | 10 |
| USK | 12000 | 0.273 | 0.05 | 8 |
